# Supplementary material for: Controlling protein stability with SULI, a highly sensitive tag for stabilization upon light induction
Source: Nat Commun. 2023 Apr 15;14:2172. doi: 10.1038/s41467-023-37830-0 (PMC10105765; doi:10.1038/s41467-023-37830-0)
Supplement: Supplementary file 1 — Supplementary information [file 41467_2023_37830_MOESM1_ESM.pdf]

SUPPLEMENTARY INFORMATION

Controlling protein stability with SULI, a highly sensitive tag of stabilization upon light induction

Miaowei Mao, Yajie Qian, Wenyao Zhang, Siyu Zhou, Zefeng Wang, Xianjun Chen, Yi Yang

Supplementary Figures

Figure 1: The effect of different LOV-containing photosensitive domains on the stability of fusion protein **2**
Figure 2: The effect of blue light on the mRNA levels of mCherry and mCherry-VVD **3**
Figure 3: The effects of different VVD variants on the stability of mCherry reporter **4**
Figure 4: The effects of combinational mutations of VVD on their light-induced stabilization of mCherry reporter **5**
Figure 5: FACS analysis of the changes in stabilities between dark and light conditions for different VVD variants **6**
Figure 6: The effects of combinational mutations of VVD on their light-induced stabilization of sfGFP reporter **7**
Figure 7: The effects of combinational mutations of VVD on their light-induced stabilization of sfGFP reporter **8**
Figure 8: Validation of the oligomerization of SUMO-VVD fusions **9**
Figure 9: Comparison of light-induced stabilization of mCherry reporter by SULI and $SULI_f$ **10**
Figure 10: Light-induced stabilization of mCherry reporter by SULI containing different mutations of Lys→Arg **11**
Figure 11: Immunoblotting analysis of light-induced stabilization of mCherry by SULI **12**
Figure 12: Light-induced stabilization of mCherry in nucleus and mitochondria by SULI **13**
Figure 13: The kinetics of SULI-mediated degradation of mCherry protein **14**
Figure 14: The Role of the Hsp104 protein in SULI-regulated protein stability **15**
Figure 15: Optical control of cell cycle **16**
Figure 16: Immunoblotting analysis of light-induced stabilization of $^{\Delta N}$Sic1 and Clb2(Δde) by SULI **17**
Figure 17: Little interference of the intrinsic fluorescence of FAD with the GFP detection **18**
Figure 18: Combinational use of SULI and yLightOn system **19**
Figure 19: The effect of light illumination on yeast growth and SULI-controlled protein stability **20**
Figure 20: The performance of SULI in regulating protein stability in mammalian cells **21**

Supplementary Tables

Table 1: Parameters for adduct decay kinetics in VVD variants **22**
Table 2: Yeast strains used in this study **23**
Table 3: Primer SULI for knock-in and qRT-PCR **24**

Supplementary Notes

Supplementary Note 1 Uncropped images of the western blots and SDS PAGE **25**
Supplementary Note 2 The amino acids sequence of SULI and $SULI_f$ **26**

Supplementary references **26**

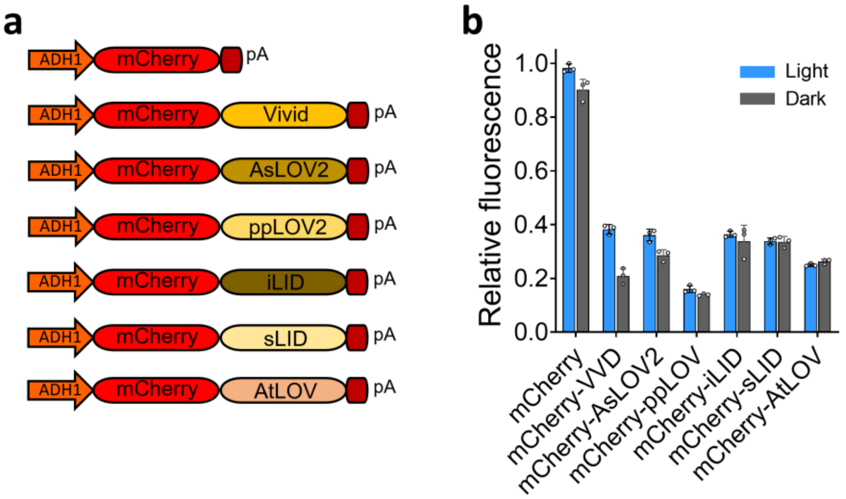

Supplementary Figure 1. The effect of different LOV-containing photosensitive domains on the stability of fusion protein. (a) Schematic representation of constructs expressing different LOV domains fused mCherry protein from ADH1 promoter. **(b)** The effect of different LOV-containing photosensitive domains on the stability of mCherry protein. Yeast cells expressing different LOV domains fusions were cultured in light or dark conditions. mCherry fluorescence of the cells was determined by flow cytometry. Data are presented as the mean ± SD from three biological replicates. Source data are provided as a Source Data file.

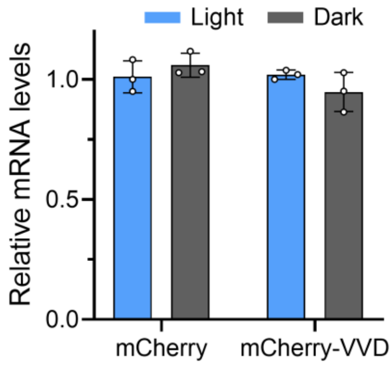

Supplementary Figure 2. The effect of blue light on the mRNA levels of mCherry and mCherry-VVD. Yeast cells expressing mCherry or mCherry-VVD were cultured under light or dark conditions for 10 h. The cells were lysed and the total RNA was extracted for analysis using quantitative PCR. Data are presented as the mean ± SD from three biological replicates. Source data are provided as a Source Data file.

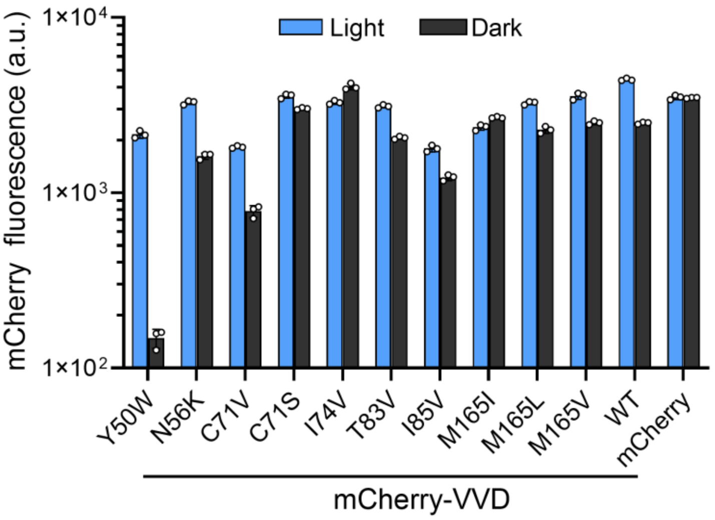

Supplementary Figure 3. The effects of different VVD variants on the stability of mCherry reporter. Yeast cells expressing mCherry-VVD fusion protein with different mutations in VVD domain were cultured under light or dark conditions for 10 h before mCherry fluorescence was determined by flow cytometry. Yeast cells expressing mCherry were used as the control. a.u., arbitrary units. Data are presented as the mean ± SD from three biological replicates. Source data are provided as a Source Data file.

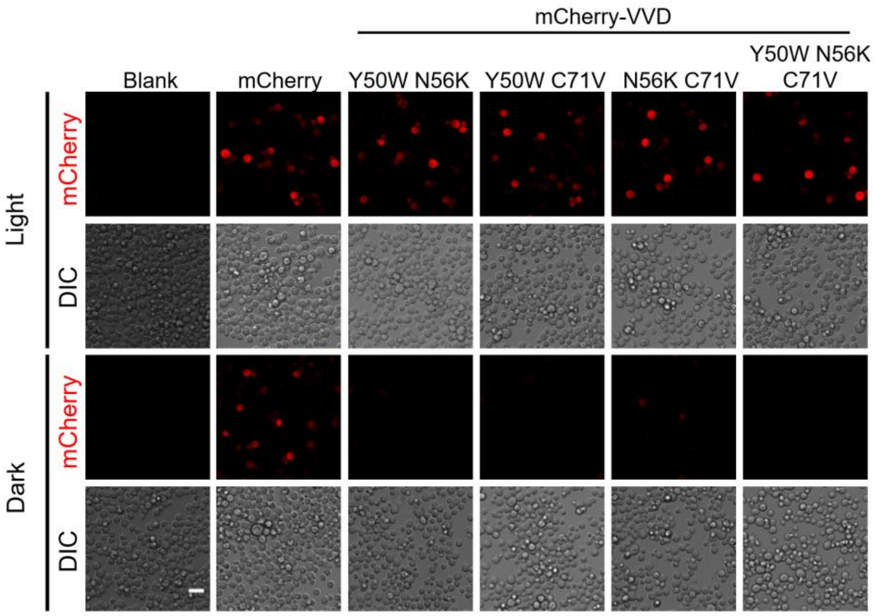

Supplementary Figure 4. The effects of combinational mutations of VVD on their light-induced stabilization of mCherry reporter. Yeast cells expressing mCherry-VVD with combinational mutations on VVD domain were cultured under light or dark conditions for 15 h before images were taken. Yeast cells expressing mCherry or none were used as the controls. Scale bar, 10 µm.

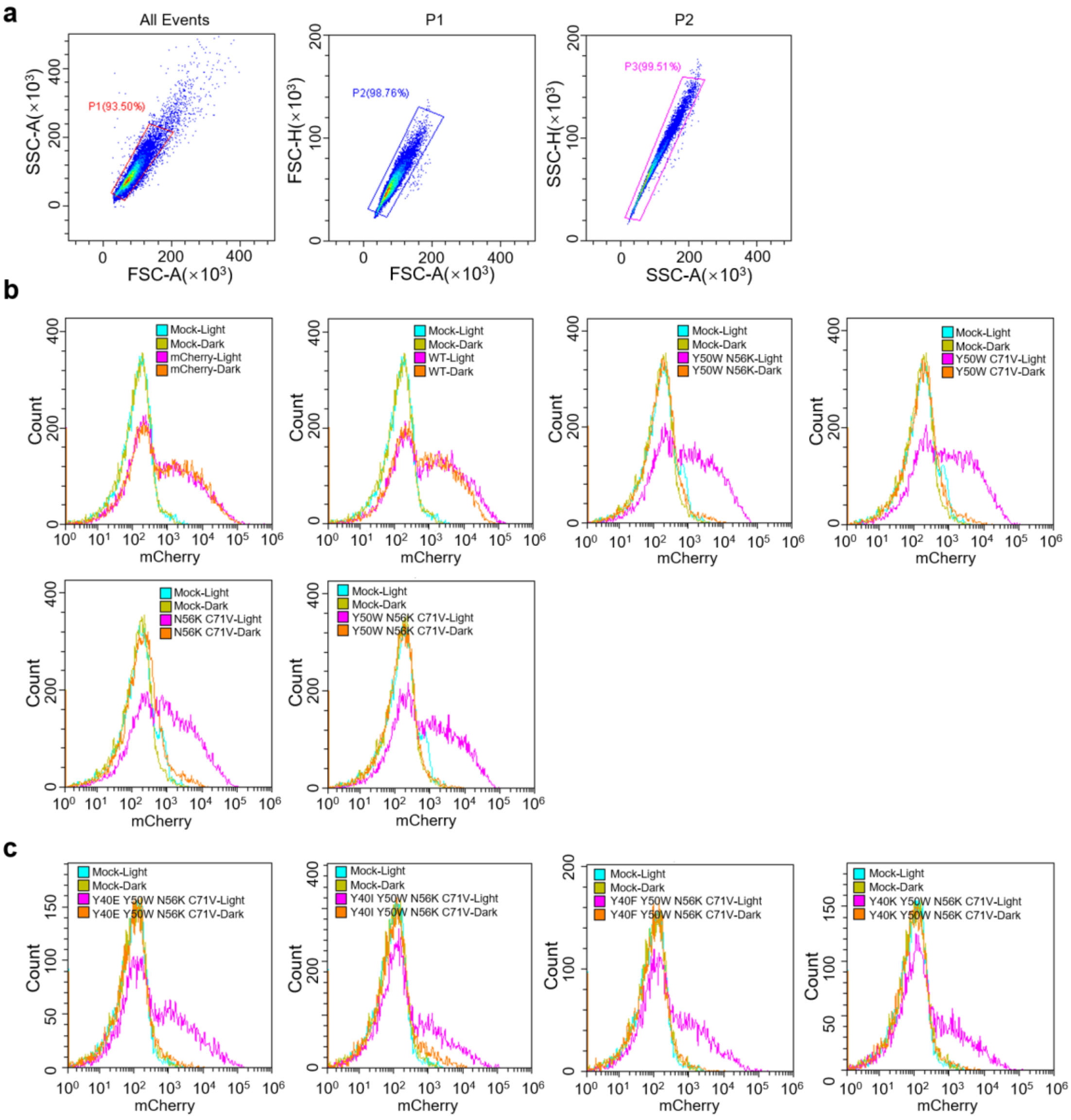

Supplementary Figure 5. FACS analysis of the changes in stabilities between dark and light conditions for different VVD variants. **(a)** The gating strategy for the FACS analysis for yeast cells in this study. **(b)** Changes in stabilities between dark and light conditions for different VVD variants. Yeast cells expressing different mCherry-VVD variants or mCherry alone were cultured under light or dark conditions. mCherry fluorescence was analyzed by flow cytometry with a 561-nm laser excitation. Yeast cells transformed with empty plasmid were used as the controls. **(c)** Effects of the Y40 mutation in VVD (Y50W N56K C71V) on light-induced stabilization. Yeast cells expressing different mCherry-VVD variants were cultured under light or dark conditions. mCherry fluorescence was analyzed using flow cytometry with a 561-nm laser excitation.

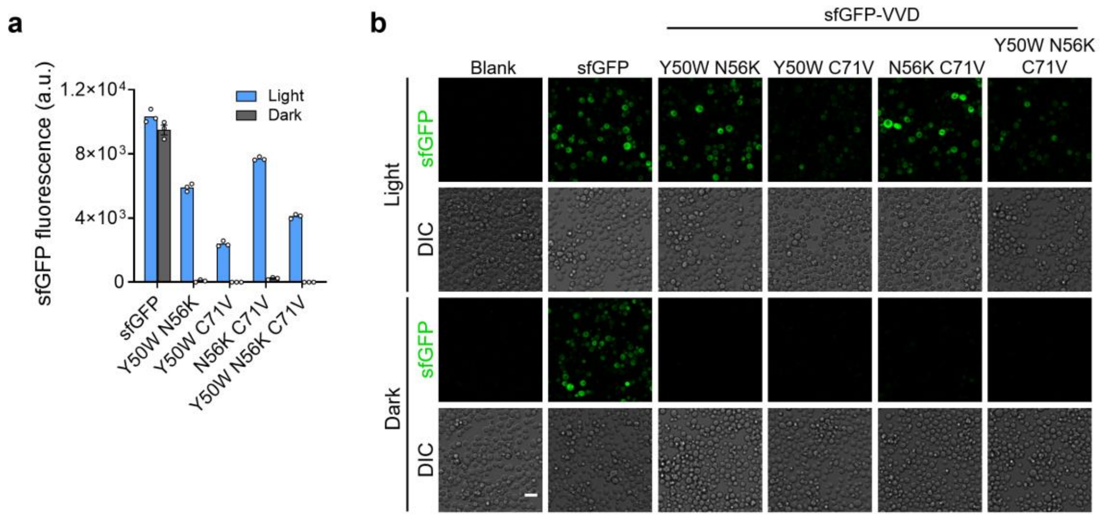

Supplementary Figure 6. The effects of combinational mutations of VVD on their light-induced stabilization of sfGFP reporter. Yeast cells expressing sfGFP-VVD with combinational mutations in VVD domain were cultured under light or dark conditions for 15 h before sfGFP fluorescence was measured by a microplate reader **(a)** and imaged **(b)**. Yeast cells expressing sfGFP or none were used as the controls. Data in **(a)** are presented as the mean ± SD from three biological replicates. a.u., arbitrary units. Scale bar in **(b)**, 10 μm. Source data are provided as a Source Data file.

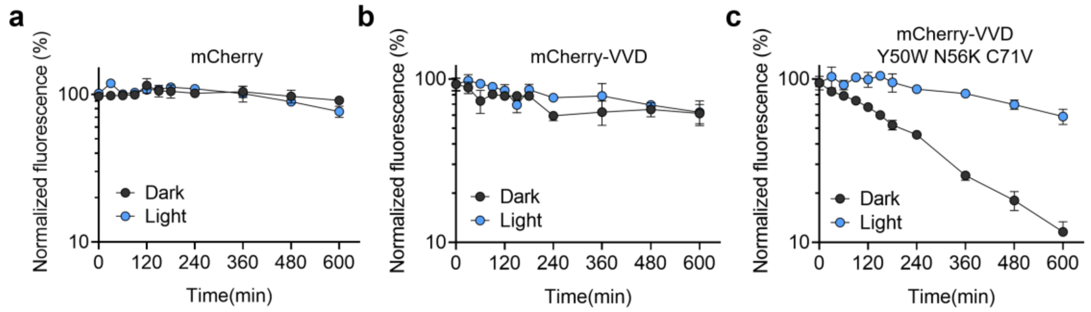

Supplementary Figure 7. Comparison of the degradation kinetics of mCherry-VVD fusions by a CHX chase experiment. Yeast cells expressing mCherry **(a)**, mCherry-VVD **(b)** or mCherry-VVD (Y50W N56K C71V) **(c)** were first cultured upon blue light illumination. The cells were incubated with cycloheximide (CHX) and transferred to dark conditions. The mCherry fluorescence at the indicated time points was determined by flow cytometry. Data are presented as the mean ± SD from three biological replicates. Source data are provided as a Source Data file.

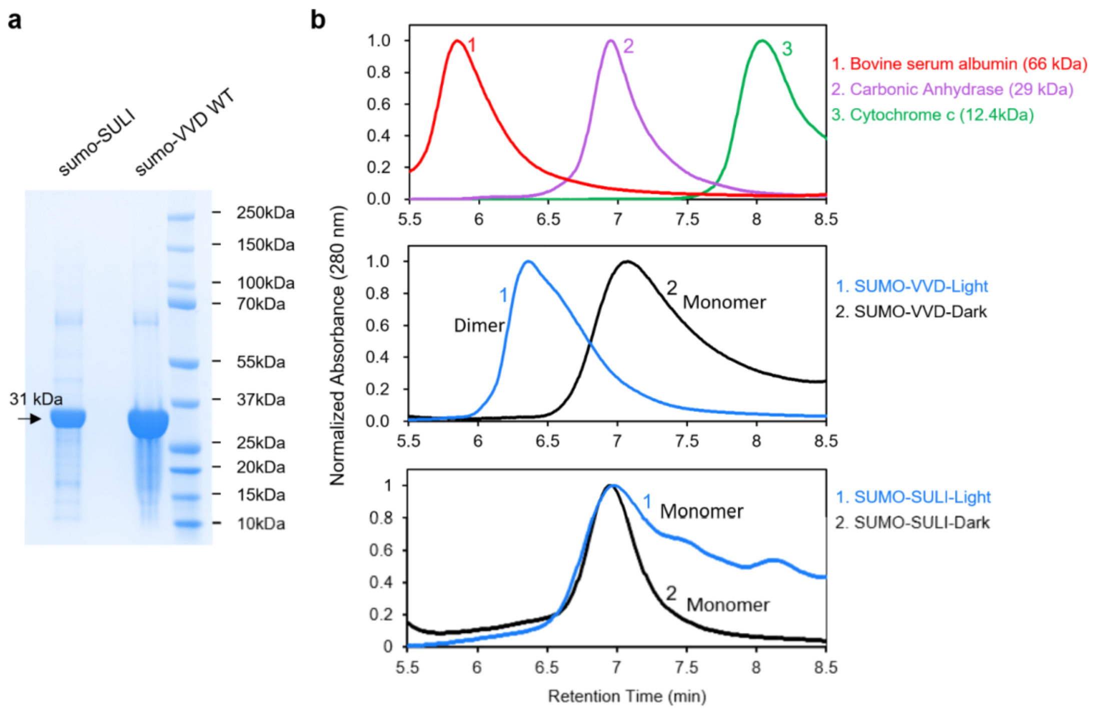

Supplementary Figure 8. Validation of the oligomerization of SUMO-VVD fusions by HPLC. **(a)** Validation of the purity of the recombinant SUMO-VVD and SUMO-SULI by SDS PAGE. **(b)** The purified SUMO-VVD and SUMO-SULI in light or dark state were analyzed by HPLC. The Bovine serum albumin (66 kDa), Carbonic Anhydrase (29 kDa) and Cytochrome c (12.4kDa) were used as the markers. The small peaks after the main peak of the monomeric state of SUMO-SULI might be the impure proteins shown in the SDS PAGE. Source data are provided as a Source Data file.

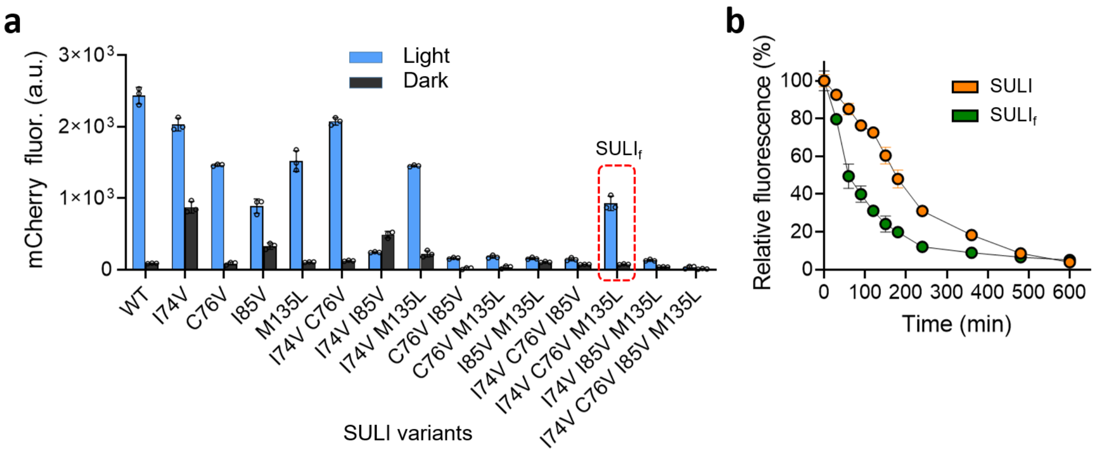

Supplementary Figure 9. Comparison of light-induced stabilization of mCherry reporter by SULI and SULI$_f$. (a) Light-induced stabilization of mCherry reporter by SULI variants containing different mutations. Yeast cells expressing different fusions were cultured under light or dark conditions for 15 h before mCherry fluorescence was determined by flow cytometry. a.u., arbitrary units. **(b)** Comparison of the degradation kinetics of mCherry-SULI$_f$ and mCherry-SULI by a CHX chase experiment. Yeast cells expressing mCherry-SULI$_f$ or mCherry-SULI were first cultured upon blue light illumination. The cells were incubated with cycloheximide (CHX) and transferred to dark conditions. The mCherry fluorescence at the indicated time points was determined by flow cytometry. Data in **(a)** and **(b)** are presented as the mean ± SD from three biological replicates. Source data are provided as a Source Data file.

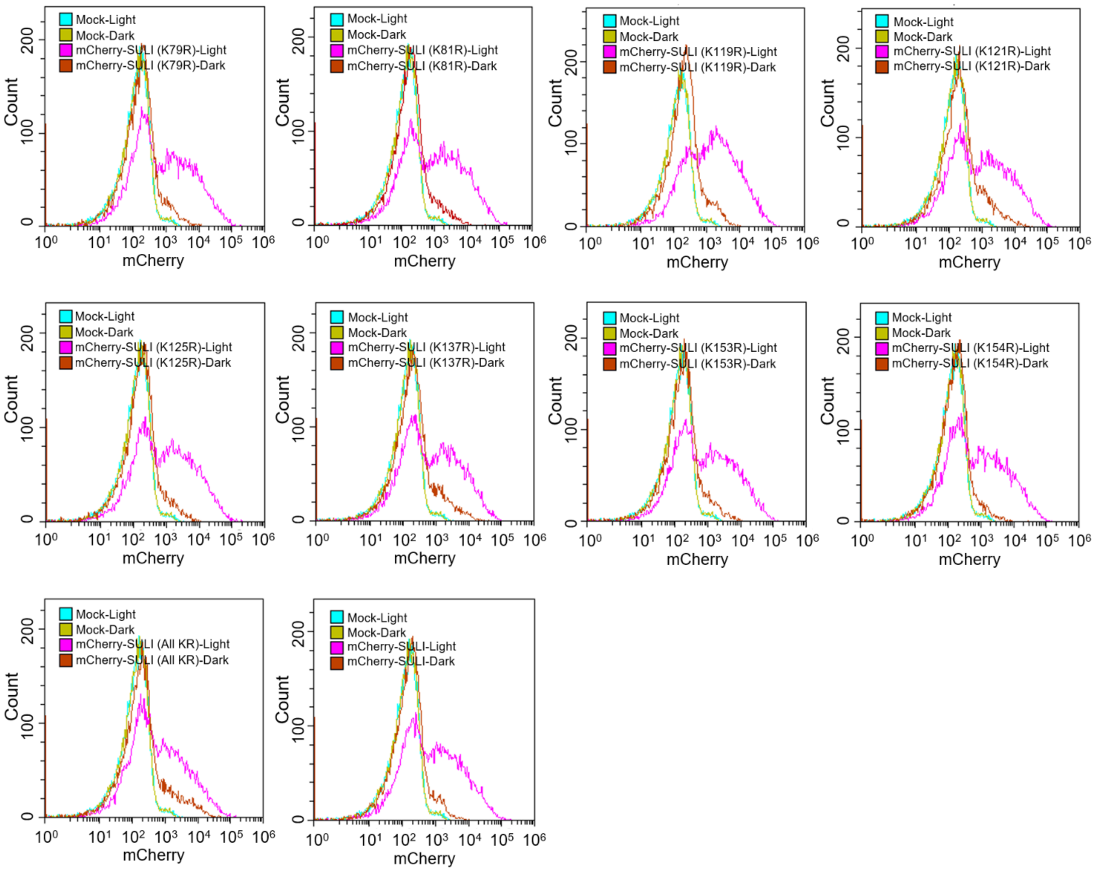

Supplementary Figure 10. Light-induced stabilization of mCherry reporter by SULI-containing different mutations of Lys to Arg. Yeast cells expressing the corresponding fusion proteins cultured under light or dark conditions were studied using flow cytometry with a 561-nm laser excitation for mCherry fluorescence. “All” means that all Lys residues in SULI were mutated to Arg. Yeast cells transformed with empty plasmid were used as the controls.

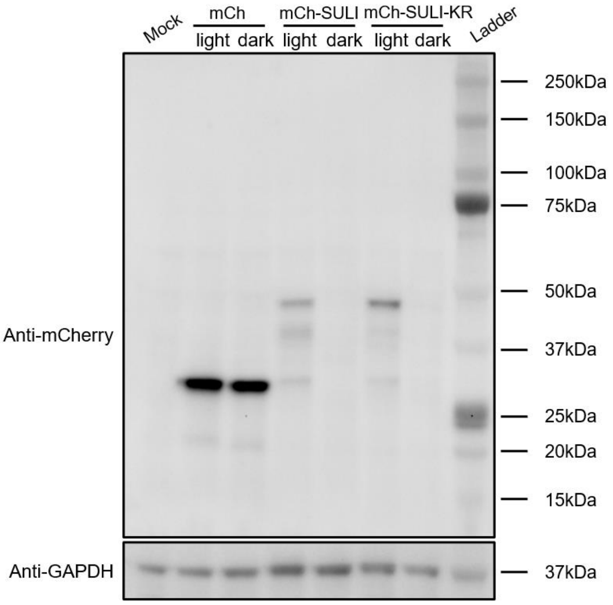

Supplementary Figure 11. Immunoblotting analysis of light-induced stabilization of mCherry by SULI. Yeast cells expressing mCherry, mCherry-SULI or mCherry-SULI KR were cultured under light or dark conditions for 10 h. The cells were lysed and analyzed using Western blot. Notably, there was discrepancy across the data set regarding to the relative expression levels of mCherry and mCherry-SULI produced with WB and fluorescence measurement, respectively. It should be aware that the data produced with western blot is typically considered to be semi-quantitative, as there are many factors that can affect the signals of target protein, e.g., antibody affinity, transfer efficiency, protein configuration, linear range and internal loading control [1, 2]. Thus, it is not recommended to precisely quantify the levels of target proteins just according to the signals of the bands, especially when the target proteins have different sequences, molecular weights, and configurations. Source data are provided as a Source Data file.

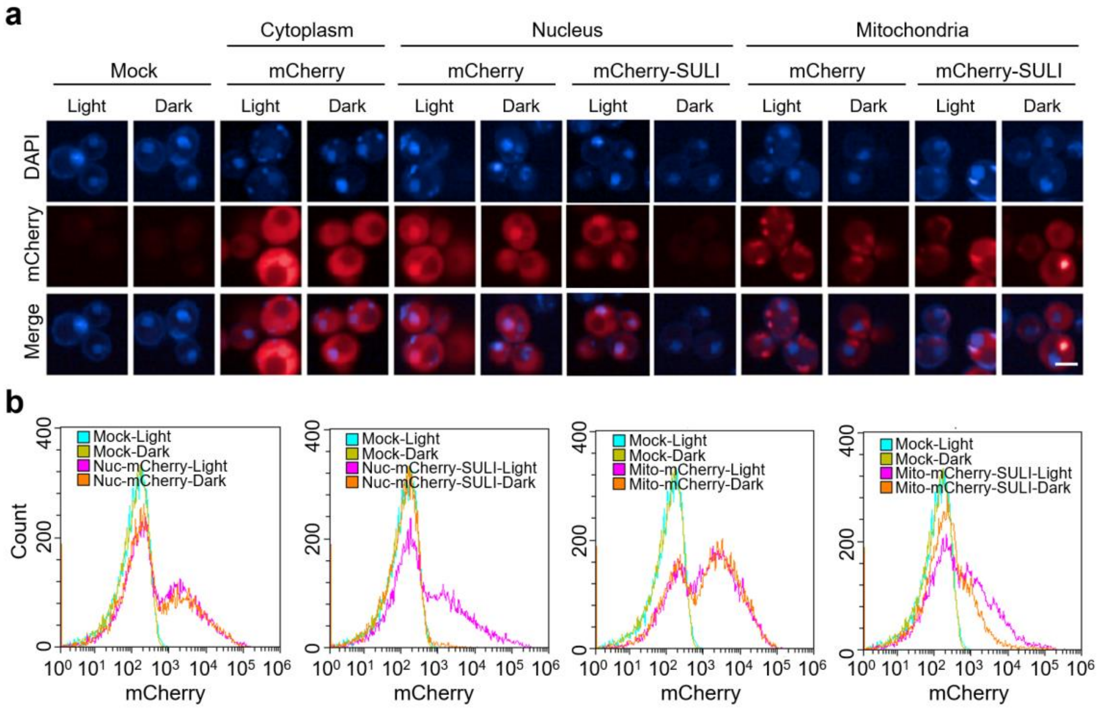

Supplementary Figure 12. Light-induced stabilization of mCherry in nucleus and mitochondria by SULI. **(a)** Yeast cells expressing nucleus or mitochondria localized mCherry-SULI were cultured under light or dark conditions for 15 h before mCherry fluorescence was imaged. Nucleus, mitochondria, or cytoplasm localized mCherry was used as the controls. The nucleus was stained with DAPI (blue). Scale bar, 5 µm. **(b)** Analysis of mCherry fluorescence by FACS. The mCherry fluorescence of the cells in **(a)** was analyzed by FACS using a 561-nm laser excitation. Yeast cells transformed with empty plasmid were used as the controls.

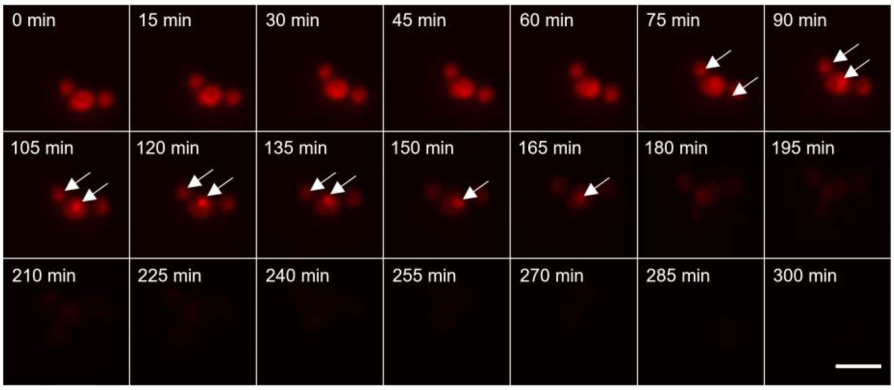

Supplementary Figure 13. The kinetics of SULI-mediated degradation of mCherry protein. Yeast cells expressing mCherry-SULI were first cultured upon illumination with blue light and then were transferred to dark conditions. The cellular mCherry fluorescence at the indicated time points was imaged after the cells were transferred to dark conditions. Scale bar, 5 µm.

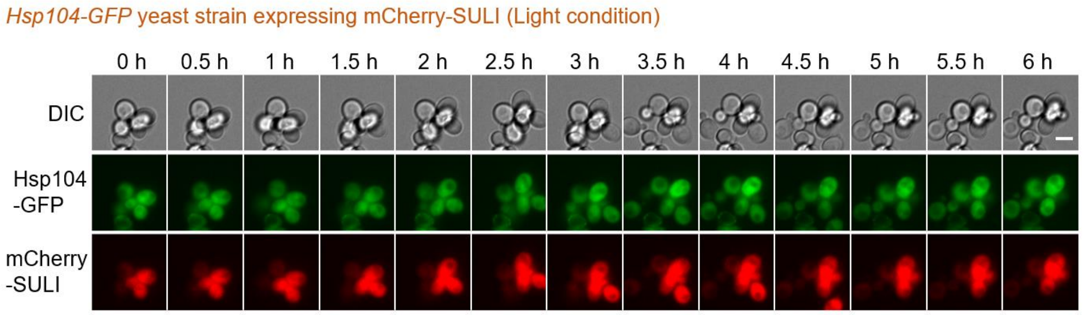

Supplementary Figure 14. The Role of the Hsp104 protein in SULI-regulated protein stability.

Yeast strains stably expressing Hsp104-GFP were used to express mCherry-SULI. The mCherry fluorescence of the cells kept in light conditions was imaged. Scale bar, 10 µm.

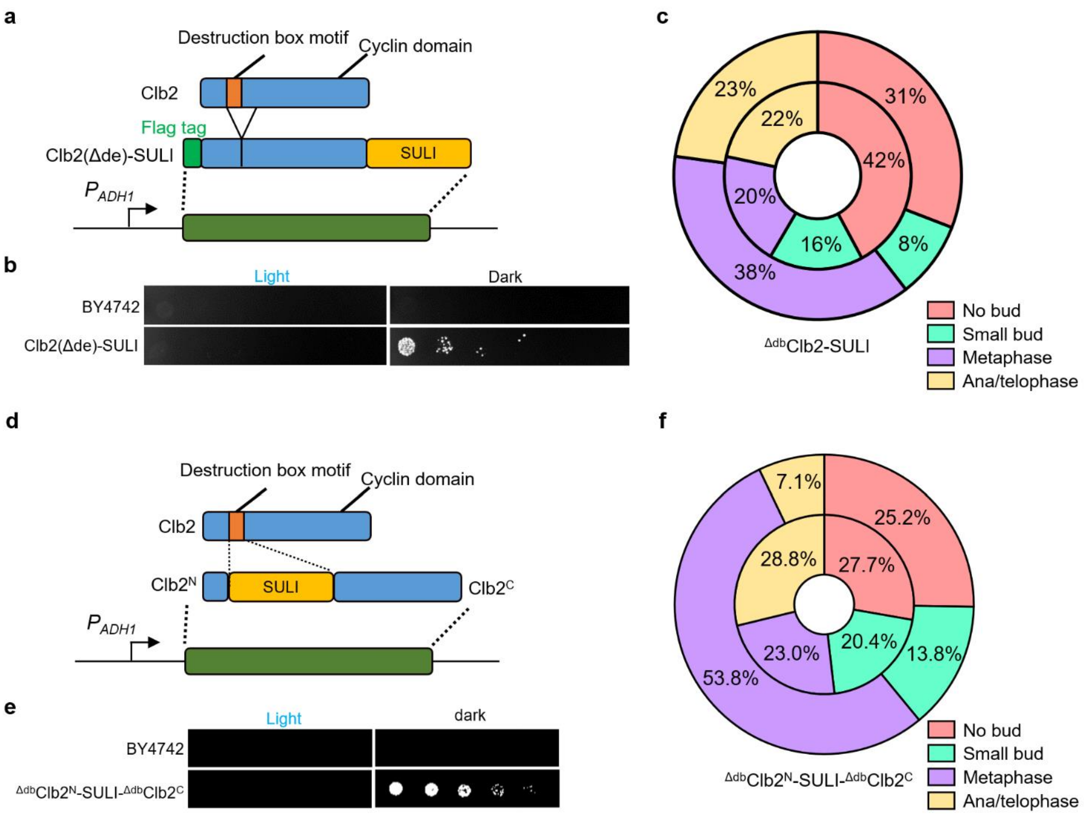

Supplementary Figure 15. Optical control of cell cycle. **(a, d)** Schematic representation of SULI-mediated degradation of Clb2(Δde). SULI was fused to the C-terminal or inner position of Clb2(Δde), a shortened variant of Clb2 lacking a destruction box motif (24-34 amino acids) for ubiquitin-mediated degradation by the proteasome. **(b, e)** Yeast cells expressing Clb2(Δde)-SULI or Clb2(Δde)^{N}-SULI-Clb2(Δde)^{C} were serially diluted (1:10; first spot approximately 2.5x10^{4} cells) and grown in solid medium under light or dark conditions. The BY4742 cells transformed with empty vectors were used as the controls. **(c, f)** The same yeast cells as in (**b**) or **(e)** were cultured under light or dark conditions for 10 h before the morphology was imaged. The distribution of cell cycle stages was analyzed from three biological replicates with at least 100 cells for each replicate.

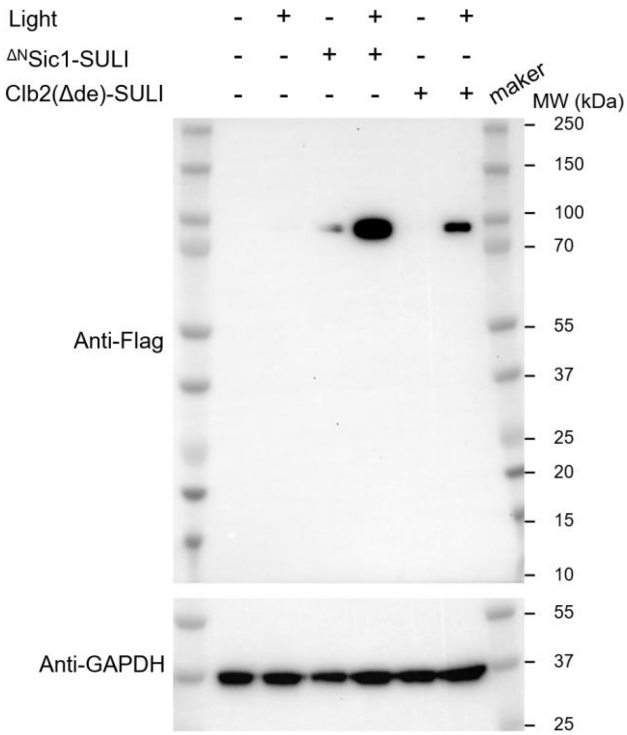

Supplementary Figure 16. Immunoblotting analysis of light-induced stabilization of $^{\Delta N}$Sic1 and Clb2(Δde) by SULI. Yeast cells expressing flag-$^{\Delta N}$Sic1-SULI and flag-Clb2(Δde)-SULI were cultured under light or dark conditions for 10 h. The cells were lysed and analyzed using Western blot. Notably, $^{\Delta N}$Sic1-SULI appeared to have a molecule weight between 70 kDa and 100 kDa, which was significantly larger than 40.1 kDa according to its amino acid sequence. It seemed that the shifted band was the phosphorylated form of $^{\Delta N}$Sic1-SULI, as the endogenous Sic1 needs to be phosphorylated at multiple sites for ubiquitination-driven degradation [3, 4]. Moreover, the phosphorylated form of a protein is typically significantly heavier than its nonphosphorylated counterpart [4, 5]. Source data are provided as a Source Data file.

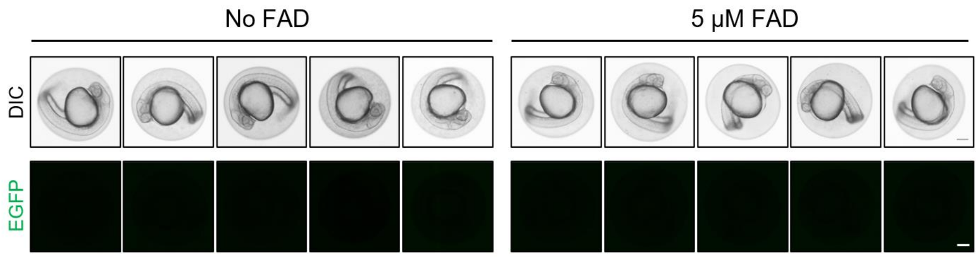

Supplementary Figure 17. Little interference of the intrinsic fluorescence of FAD with the GFP detection. The zebrafish embryos injected with empty plasmid were incubated in medium with or without 5 μM FAD for 24 h before the fluorescence at the GFP channel was imaged. Scale bar, 200 μm.

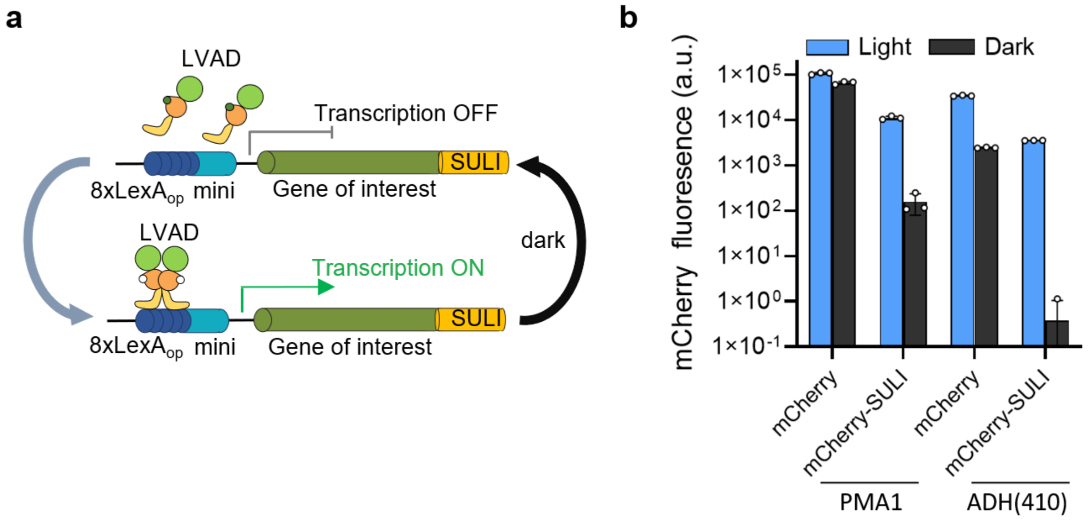

Supplementary Figure 18. Combinational use of SULI and yLightOn system. (a) Schematic of the combinational use of SULI and yLightOn system. mCherry reporter was fused with SULI and placed under the control of LVAD light-switchable transactivator. Under dark conditions, the leak expression of mCherry-SULI by yLightOn was degraded by SULI, thus reducing the leakage of reporter in the uninduced states. **(b)** Yeast cells expressing mCherry-SULI driven by yLightOn system were cultured under light or dark conditions for 15 h before mCherry fluorescence was measured by flow cytometry. The LVAD light-switchable transactivator were expressed from a PMA1 strong promoter or a ADH (410) weak promoter. a.u., arbitrary units. Data are presented as the mean ± SD from three biological replicates. Source data are provided as a Source Data file.

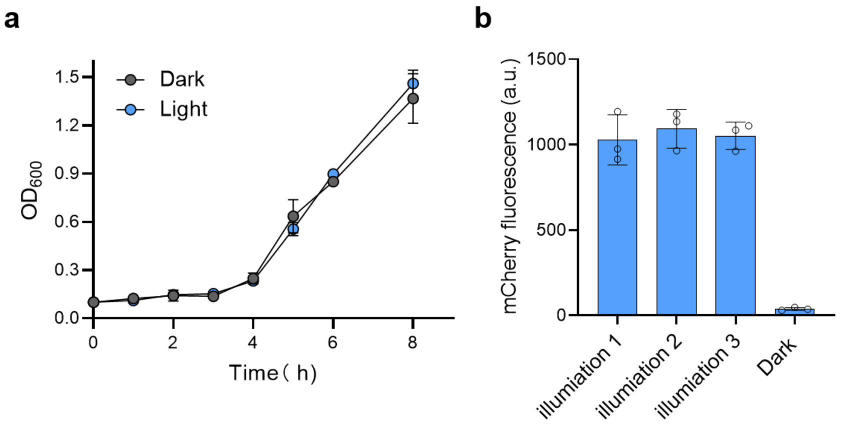

Supplementary Figure 19. The effect of light illumination on yeast growth and SULI-controlled protein stability. (a) The effect of light illumination on yeast growth. Yeast cells expressing mCherry-SULI were cultured under 15 µmol m^{-2} s^{-1} blue light illumination or dark conditions and the OD_{600} was measured at the indicated time points. Data are presented as the mean ± SD from three biological replicates. **(b)** Activation of SULI by pulsed illumination with different repetitive frequencies. Yeast cells expressing mCherry-SULI were cultured under continuous or pulsed illumination of 15 µmol m^{-2} s^{-1} blue light or dark conditions for 15 h before mCherry fluorescence was determined using by flow cytometry. Illumination 1, continuous illumination; illumination 2, pulsed illumination with 1 s of light and 9 s of dark; illumination 3, pulsed illumination with 1 s of light and 29 s of dark. The a.u. in vertical axis means arbitrary units. Data are presented as the mean ± SD from three biological replicates. Source data are provided as a Source Data file.

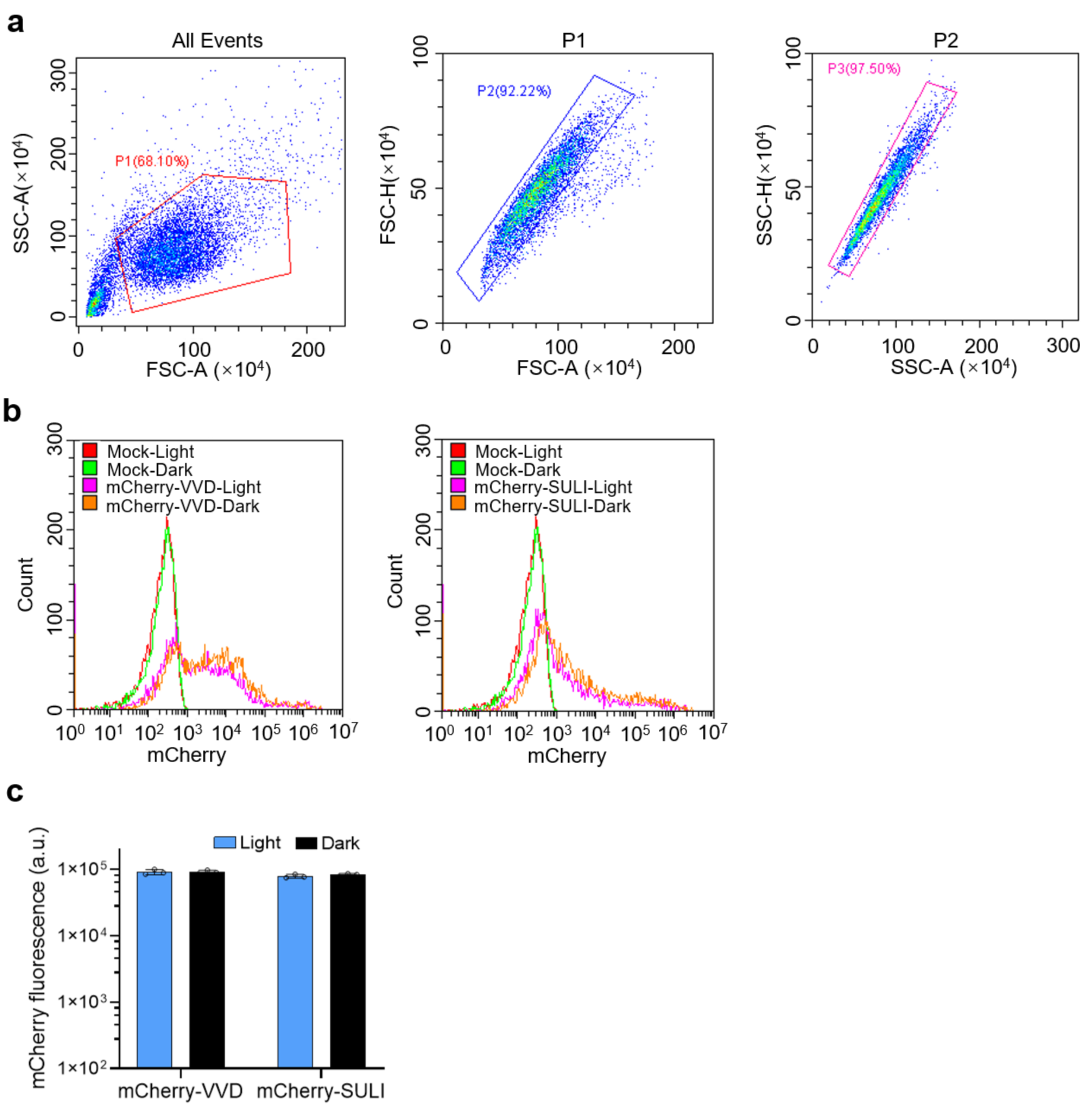

Supplementary Figure 20. The performance of SULI in regulating protein stability in mammalian cells. (a) The gating strategy for the FACS analysis. HEK293T cells coexpressing EGFP-Hsp104 and mCherry-VVD or mCherry-SULI were cultured under light or dark conditions for 24 h before mCherry fluorescence was measured by flow cytometry. **(b)** FACS analysis of mCherry fluorescence for the cells cultured under light or dark conditions. **(c)** Quantitative analysis of mCherry fluorescence in the cells coexpressing EGFP-Hsp104 and mCherry-VVD or mCherry-SULI. The a.u. in vertical axis means arbitrary units. Data are presented as the mean ± SD from three biological replicates. Source data are provided as a Source Data file.

Supplementary Table 1. Parameters for adduct decay kinetics in VVD variants

Variant	Lifetime (sec)	Rate constant (s^{-1})	kb_{cat} (s^{-1})
VVD-36	18,000	5.6×10^{-5}	6.1×10^{-4}
I74V	730	1.4×10^{-3}	6.8×10^{-5}
I85V	780	1.3×10^{-3}	3.0×10^{-3}
C76V	21,000	4.8×10^{-5}	4.1×10^{-4}
M135L	23,000	4.3×10^{-5}	4.8×10^{-4}

All data were collected from Zoltowski et al [6].

Supplementary Table 2. Yeast strains used in this study

Name	Genotype	Source	Reference
BY4742	MATα his3Δ1 leu2Δ0 lys2Δ0 ura3Δ0	Dai Lab	Baker Brachmann, Carrie, et al. Yeast 14.2 (1998): 115-132.
Erg6 knockout	BY4742 MATα his3Δ1 leu2Δ0 lys2Δ0 ura3Δ0 erg6::kanMX	Dai Lab	Winzeler, Elizabeth A., et al. *science* 285.5429 (1999): 901-906.
ADE2-mCherry knock-in	BY4742 ade2-Flag-mCherry::LEU2	ADE2 knock-in strains, this work	
ADE2-mCherry-SULI knock-in	BY4742 ade2-Flag-SULI::LEU2	ADE2 knock-in strains, this work	

Supplementary Table 3. Primer SULI for knock-in and qRT-PCR

Name	Sequence (5'-3')	Note
mCherry-F(Flag)	TTATAAAGATGATGACGATAAAGGATCCGCTATGGTG AGCAAGGGCGAGGAGG	For integration of exogenous gene into the yeast genome
Flag-Vivid-F	TCCAGATCTGATTATAAAGATGATGACGATAAAGAAT TCGGTTCTCATACGCTC	
pG-R	GGCAAAACGATGTATAAATGAAAG	
Flag-mCherry-F(ADE2）	AAAGCACAAAAGTTAGAAACTGTCGGTTACGAAGCTT ATCTAGAAAACAAGTCCAGATCTGATTATAAAGATGA TG	
LEU-R(ADE2）	ATATCATTTTATAATTATTTGCTGTACAAGTATATCAAT AAACTTATATAAACTGTGGGAATACTCAGGTATCG	
ADE2-seq-F	GGAGTAGATTCTTTACATTCAATTGTGC	
ADE2-3UTR-R	GCTATCCTCGGTTCTGCATTGAG	
mCherry-F	CTTCAAGTGGGAGCGCGTG	For RT-qPCR
mCherry-R	CAGCCTCTGCTTGATCTCGCC	
GAPDH-F	TTGCCATCAATGACCCCTTCA	
GAPDH-R	CGCCCCACTTGATTTTGGA	

Supplementary Note

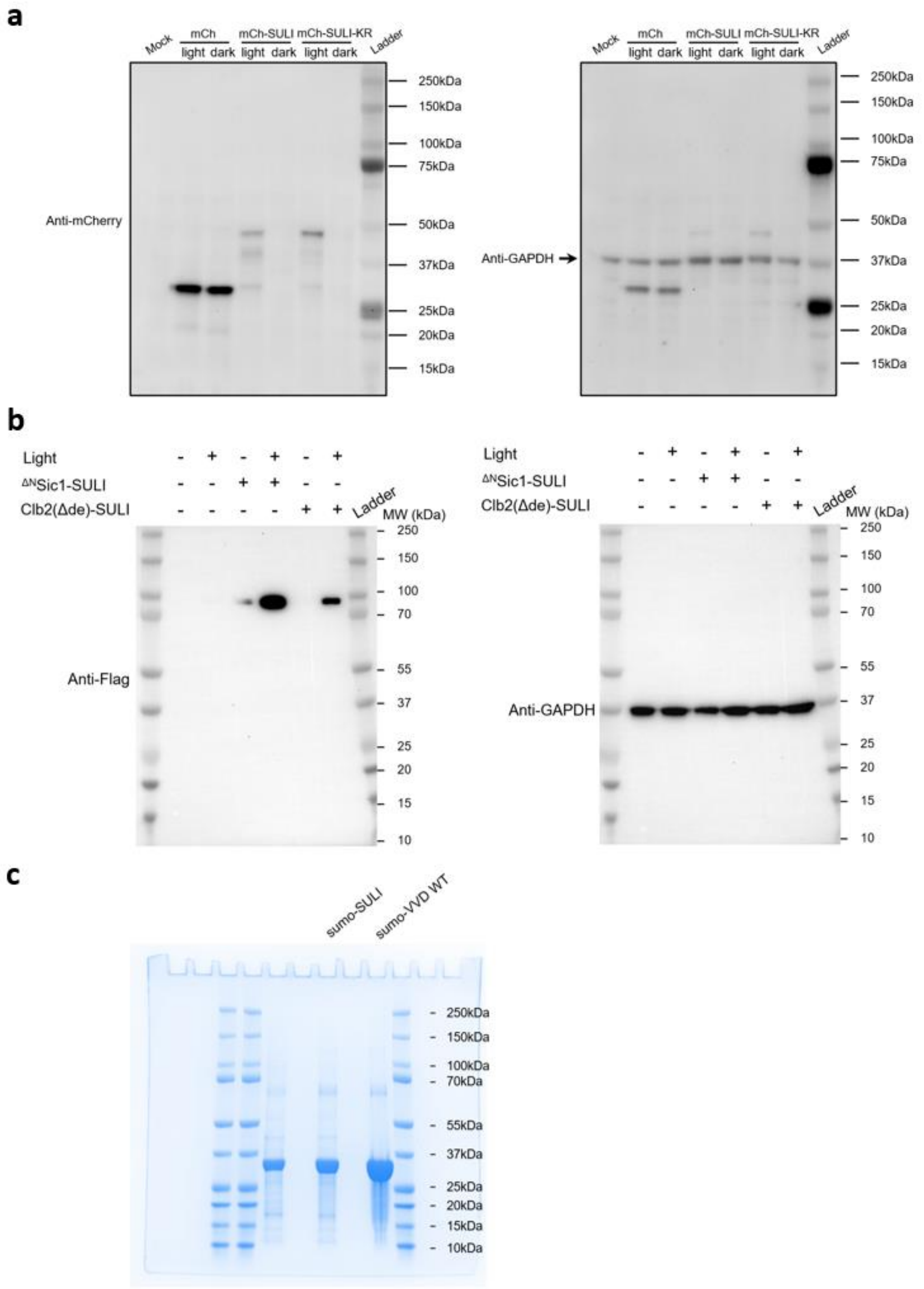

Supplementary Note 1 Uncropped images of the western blots and SDS PAGE. (a) The uncropped images of the western blot presented in **Supplementary Fig. 11**. **(b)** The uncropped images of the western blot presented in **Supplementary Fig. 16**. **(c)** The uncropped image of the SDS-PAGE gel presented in **Supplementary Figure 8a**.

Supplementary Note 2 The amino acids sequence of SULI and $SULI_f$

The amino acids sequence of SULI

HTLEAPGGYDIMGWLIQIMKRPNPQVELGPVDTSVALILCDLKQKDTPIVYASEAFLYMTGYSNAEVLGRN
CRFLQSPDGMVKPKSTRKYVDSNTINTMRKAIDRNAEVQVEVVNFKKNGQRFVNFLTMIPVRDETGEYR
YSMGFQCETE

The amino acids sequence of $SULI_f$

HTLEAPGGYDIMGWLIQIMKRPNPQVELGPVDTSVALVLVDLKQKDTPIVYASEAFLYMTGYSNAEVLGR
NCRFLQSPDGMVKPKSTRKYVDSNTINTLRKAIDRNAEVQVEVVNFKKNGQRFVNFLTMIPVRDETGEYR
YSMGFQCETE

Supplementary references

1. Pillai-Kastoori, L., Schutz-Geschwender, A.R. & Harford, J.A. A systematic approach to quantitative Western blot analysis. *Analytical biochemistry* **593**, 113608 (2020).
2. McDonough, A.A., Veiras, L.C., Minas, J.N. & Ralph, D.L. Considerations when quantitating protein abundance by immunoblot. *American journal of physiology. Cell physiology* **308**, C426-433 (2015).
3. Nash, P. et al. Multisite phosphorylation of a CDK inhibitor sets a threshold for the onset of DNA replication. *Nature* **414**, 514-521 (2001).
4. Moreno-Torres, M., Jaquenoud, M., Peli-Gulli, M.P., Nicastro, R. & De Virgilio, C. TORC1 coordinates the conversion of Sic1 from a target to an inhibitor of cyclin-CDK-Cks1. *Cell discovery* **3**, 17012 (2017).
5. Venta, R. et al. A processive phosphorylation circuit with multiple kinase inputs and mutually diversional routes controls G1/S decision. *Nature communications* **11**, 1836 (2020).
6. Zoltowski, B.D., Vaccaro, B. & Crane, B.R. Mechanism-based tuning of a LOV domain photoreceptor. *Nature chemical biology* **5**, 827-834 (2009).
