# Supplementary material for: Controlling protein stability with SULI, a highly sensitive tag for stabilization upon light induction
Source: Nat Commun. 2023 Apr 15;14:2172. doi: 10.1038/s41467-023-37830-0 (PMC10105765; doi:10.1038/s41467-023-37830-0)
Supplement: Supplementary file 3 — Reporting Summary [file 41467_2023_37830_MOESM3_ESM.pdf]

nature portfolio

Corresponding author(s): Yi Yang

Last updated by author(s): Mar 28, 2023

Reporting Summary

Nature Portfolio wishes to improve the reproducibility of the work that we publish. This form provides structure for consistency and transparency in reporting. For further information on Nature Portfolio policies, see our Editorial Policies and the Editorial Policy Checklist.

Statistics

For all statistical analyses, confirm that the following items are present in the figure legend, table legend, main text, or Methods section.

n/a	Confirmed	
☐	☒	The exact sample size (*n*) for each experimental group/condition, given as a discrete number and unit of measurement
☐	☒	A statement on whether measurements were taken from distinct samples or whether the same sample was measured repeatedly
☐	☒	The statistical test(s) used AND whether they are one- or two-sided<br>*Only common tests should be described solely by name; describe more complex techniques in the Methods section.*
☒	☐	A description of all covariates tested
☒	☐	A description of any assumptions or corrections, such as tests of normality and adjustment for multiple comparisons
☐	☒	A full description of the statistical parameters including central tendency (e.g. means) or other basic estimates (e.g. regression coefficient) AND variation (e.g. standard deviation) or associated estimates of uncertainty (e.g. confidence intervals)
☐	☒	For null hypothesis testing, the test statistic (e.g. *F*, *t*, *r*) with confidence intervals, effect sizes, degrees of freedom and *P* value noted<br>*Give P values as exact values whenever suitable.*
☒	☐	For Bayesian analysis, information on the choice of priors and Markov chain Monte Carlo settings
☒	☐	For hierarchical and complex designs, identification of the appropriate level for tests and full reporting of outcomes
☒	☐	Estimates of effect sizes (e.g. Cohen's *d*, Pearson's *r*), indicating how they were calculated

Our web collection on statistics for biologists contains articles on many of the points above.

Software and code

Policy information about availability of computer code

Data collection	A Synergy 2 multimode microplate reader (BioTek) was used for measuring fluorescence and OD600 of yeast cells. A CytoFLEX-S flow cytometer (Beckman Coulter) was used analyze the fluorescence of yeast cells. A ChemiDoc Touch Imaging System (Bio-Rad) was used for detection of chemiluminescence. A Roche LightCycle 480 Real-Time PCR System was used to collect the qPCR data. An LC-20A HPLC system (Shimadzu) was used to assay the oligomerization states of the sumo-SULI fusion proteins. A Leica SP8 confocal laser scanning microscope (LAS X Version 3.5)or a Nikon Eclipse Ti-E microscope was used for fluorescence imaging. A Nikon Eclipse Ti2 microscope was used for bioluminescence imaging of NLuc. A Tanon-5200 system was used for fluorescence imaging of yeast cells on plate. An Eclipse Ti2 inverted microscope system (Nikon) was used to analyze the cell cycle stages of yeast cells.
Data analysis	Gene 5 reader control (Gene 5 version 2.0.19.0)and data analysis software was used to process the microplate reader data. Microsoft Excel 365 and Graphpad Prism (Version 8) were used for data analysis and presentation. CytExpert (Beckman Coulter) software (version 2.3.0.84) was used to process flow cytometry data. The Image Lab software (Bio-Rad) (version 6.0.0 build 25) was used to process the data from ChemiDoc Touch Imaging System (Image Lab Touch Software, version 1.2.0.12, Bio-Rad). The LightCycler 480 Software was used to analyze the qPCR data. The Lab solution software (version 5.96 SP3, Shimadzu) was used to analyze the data from HPLC system. The ImageJ software (NIH) (version 1.47t) were used to process imaging data.

For manuscripts utilizing custom algorithms or software that are central to the research but not yet described in published literature, software must be made available to editors and reviewers. We strongly encourage code deposition in a community repository (e.g. GitHub). See the Nature Portfolio guidelines for submitting code & software for further information.

Data

Policy information about availability of data

All manuscripts must include a data availability statement. This statement should provide the following information, where applicable:

- Accession codes, unique identifiers, or web links for publicly available datasets
- A description of any restrictions on data availability
- For clinical datasets or third party data, please ensure that the statement adheres to our policy

The experimental data generated in this study are provided in the Supplementary Information/Source Data file. The constructs generated in this study are freely available upon request from the corresponding authors with appropriate Material Transfer Agreement (MTA). Source data are provided with this paper.

Human research participants

Policy information about studies involving human research participants and Sex and Gender in Research.

Reporting on sex and gender	n/a
Population characteristics	n/a
Recruitment	n/a
Ethics oversight	n/a

Note that full information on the approval of the study protocol must also be provided in the manuscript.

Field-specific reporting

Please select the one below that is the best fit for your research. If you are not sure, read the appropriate sections before making your selection.

☒ Life sciences ☐ Behavioural & social sciences ☐ Ecological, evolutionary & environmental sciences

For a reference copy of the document with all sections, see nature.com/documents/nr-reporting-summary-flat.pdf

Life sciences study design

All studies must disclose on these points even when the disclosure is negative.

Sample size	No statistical methods were used to pre-determine sample size. The sample size (n) of each experiment is provided in each figure legend in the main manuscript and supplementary information file. Following standards of the field, sample sizes were estimated which were capable of yielding statistically significant. 10,000 and 5,000 cells were used for the FACS detection in yeast and mammalian cells, respectively. The differences between groups were compared using the two-tailed student's t-test.
Data exclusions	No data was excluded from the analysis.
Replication	Each data in this manuscript is reliably reproduced. The replication number of each data is indicated in the legend of corresponding figures.
Randomization	All samples were randomly assigned into control or experimental groups.
Blinding	No blinding was performed because no studies were deemed to be influenced by human interpretation.

Reporting for specific materials, systems and methods

We require information from authors about some types of materials, experimental systems and methods used in many studies. Here, indicate whether each material, system or method listed is relevant to your study. If you are not sure if a list item applies to your research, read the appropriate section before selecting a response.

Materials & experimental systems

n/a	Involved in the study	
☐	☒	Antibodies
☐	☒	Eukaryotic cell lines
☒	☐	Palaeontology and archaeology
☐	☒	Animals and other organisms
☒	☐	Clinical data
☒	☐	Dual use research of concern

Methods

n/a	Involved in the study	
☒	☐	ChIP-seq
☐	☒	Flow cytometry
☒	☐	MRI-based neuroimaging

Antibodies

Antibodies used	*Anti-mCherry antibody [1C51] (Cat# ab125096, Abcam).<br>*Monoclonal ANTI-FLAG M2 antibody (Cat#F1804, Sigma).<br>*HRP-conjugated GAPDH Mouse mAb [AMC0500] (Cat# AC035, Abclonal).<br>*Anti-mouse IgG, HRP-linked Antibody (Cat#7076, Cell Signaling Technology).
Validation	All antibodies were verified by the manufacturers and each lot has been quality tested. All validation statements can be found on the antibody website, respectively:<br>*Anti-mCherry antibody [1C51] (Cat# ab125096, Abcam).<br>See: https://www.abcam.com/mcherry-antibody-1c51-ab125096.html<br>or https://antibodyregistry.org/search.php?q=AB_11133266<br>*Monoclonal ANTI-FLAG M2 antibody (Cat#F1804, Sigma).<br>See: https://www.sigmaaldrich.com/US/en/product/sigma/f1804<br>or https://antibodyregistry.org/search.php?q=AB_262044<br>*HRP-conjugated GAPDH Mouse mAb (Cat# AC035, Abclonal).<br>See: https://abclonal.com/catalog-antibodies/HRPconjugatedGAPDHMousemAb/AC035<br>*Anti-mouse IgG, HRP-linked Antibody (Cat#7076, Cell Signaling Technology)<br>See: https://www.cellsignal.com/products/secondary-antibodies/anti-mouse-igg-hrp-linked-antibody/7076<br>or https://antibodyregistry.org/search.php?q=AB_330924

Eukaryotic cell lines

Policy information about cell lines and Sex and Gender in Research

Cell line source(s)	BY4742, ΔHsp104 and Hsp104-GFP yeast strains were provided by Dr. Junbiao Dai (Center for Synthetic Genomics, Shenzhen Institutes of Advanced Technology, CAS). HEK 293T cell line was purchased from National Collection of Authenticated Cell Cultures, Center for Excellence in Molecular Cell Science, CAS.
Authentication	Cell lines were used without further authentication.
Mycoplasma contamination	Yeast cell lines were not susceptible to Mycoplasma contamination. No mycoplasma was detected in the HEK 293T cell line.
Commonly misidentified lines<br>(See ICLAC register)	n/a

Animals and other research organisms

Policy information about studies involving animals; ARRIVE guidelines recommended for reporting animal research, and Sex and Gender in Research

Laboratory animals	Danio rerio AB strain. One-cell-stage zebrafish embryos and 2-d zebrafish larvae. Sex was not considered in this study.
Wild animals	The study did not involve wild animals.
Reporting on sex	In this study, sex was not considered in study design.
Field-collected samples	The study did not involve samples collected from the field.
Ethics oversight	The zebrafish handling protocols were approved by the Ethical Review Committee of CAS Center for Excellence in Molecular Cell Science, Chinese Academy of Sciences (CAS), China.

Note that full information on the approval of the study protocol must also be provided in the manuscript.

Flow Cytometry

Plots

Confirm that:

☒ The axis labels state the marker and fluorochrome used (e.g. CD4-FITC).

☒ The axis scales are clearly visible. Include numbers along axes only for bottom left plot of group (a 'group' is an analysis of identical markers).

☒ All plots are contour plots with outliers or pseudocolor plots.

☒ A numerical value for number of cells or percentage (with statistics) is provided.

Methodology

Sample preparation	Unless otherwise indicated, the overnight cultured yeast cells were diluted (1:1000) into fresh medium and grown under light or dark conditions for 10 or 15 hours. For detection by flow cytometry, the yeast cells were diluted 10-fold with fresh synthetic medium in a 96-well V-bottom plate. The total volume was greater than 220 µL for safe operation. The samples were mixed and single-cell fluorescence was measured in a Beckman CytoFLEX S flow cytometry. For detection of mCherry fluorescence in mammalian cells, the transfected cells were harvested by trypsin digestion and resuspended with 200 µL PBS buffer.
Instrument	A CytoFLEX-S flow cytometer (Beckman Coulter) was used to acquire data.
Software	Data was analyzed by using Cytexpert program (Beckman Coulter) (version 2.3.0.84).
Cell population abundance	All reported populations were greater than 10,000 and 5,000 cells for the studies in yeast and mammalian cells, respectively.
Gating strategy	The gating strategy for yeast cells was: 1) FSC-A/SSC-A gate for living cells; and 2) FSC-H/FSC-A and SSC-H/SSC-A for individual cell. The gating strategy for yeast cells was: 1) FSC-A/SSC-A gate for living cells; and 2) FSC-H/FSC-A and SSC-H/SSC-A for individual cell; 3) using the mock cells to define the gate for GFP positive cells.

☒ Tick this box to confirm that a figure exemplifying the gating strategy is provided in the Supplementary Information.
